# Antimicrobial Resistance Mechanisms and Virulence of Colistin- and Carbapenem-Resistant *Acinetobacter baumannii* Isolated from a Teaching Hospital in Taiwan

**DOI:** 10.3390/microorganisms9061295

**Published:** 2021-06-14

**Authors:** Noor Andryan Ilsan, Yuarn-Jang Lee, Shu-Chen Kuo, I-Hui Lee, Tzu-Wen Huang

**Affiliations:** 1 International Master/Ph.D. Program in Medicine, College of Medicine, Taipei Medical University, Taipei 11031, Taiwan; noorandryanilsan@gmail.com; 2 Department of Microbiology and Immunology, School of Medicine, College of Medicine, Taipei Medical University, Taipei 11031, Taiwan; sos810712@gmail.com; 3 Department of Internal Medicine, Division of Infectious Diseases, Taipei Medical University Hospital, Taipei 11031, Taiwan; yuarn438@yahoo.com.tw; 4 Department of Internal Medicine, Division of Infectious Diseases, School of Medicine, College of Medicine, Taipei Medical University, Taipei 11031, Taiwan; 5 National Institute of Infectious Diseases and Vaccinology, National Health Research Institutes, Zhunan 35053, Taiwan; sckuo@nhri.edu.tw; 6 Graduate Institute of Medical Sciences, College of Medicine, Taipei Medical University, Taipei 11031, Taiwan

**Keywords:** nosocomial pathogen, whole-genome sequencing, two-component system, lipopolysaccharide, phosphoethanolamine transferase, biofilm, heteroresistance, *Galleria mellonella*

## Abstract

*Acinetobacter baumannii*, a Gram-negative bacterium, is an important nosocomial pathogen. Colistin-resistant *A. baumannii* is becoming a new concern, since colistin is one of the last-line antibiotics for infections by carbapenem-resistant *A. baumannii.* From 452 carbapenem-resistant isolates collected in a teaching hospital in Taipei, Taiwan, we identified seven that were resistant to colistin. Carbapenem resistance in these isolates is attributed to the presence of carbapenemase gene *bla*_OXA-23_ in their genomes. Colistin resistance is presumably conferred by mutations in the sensor kinase domain of PmrB found in these isolates, which are known to result in modification of colistin target lipid A via the PmrB–PmrA–PmrC signal transduction pathway. Overexpression of *pmrC*, *eptA,* and *naxD* was observed in all seven isolates. Colistin resistance mediated by *pmrB* mutations has never been reported in Taiwan. One of the seven isolates contained three mutations in *lpxD* and exhibited an altered lipopolysaccharide profile, which may contribute to its colistin resistance. No significant difference in growth rates was observed between the isolates and the reference strain, suggesting no fitness cost of colistin resistance. Biofilm formation abilities of the isolates were lower than that of the reference. Interestingly, one of the isolates was heteroresistant to colistin. Four of the isolates were significantly more virulent to wax moth larvae than the reference.

## 1. Introduction

The global increase in antimicrobial resistance (AMR) among pathogenic bacteria threatens human health. Deaths caused by antimicrobial-resistant bacteria are expected to exceed deaths caused by cancers in 2050 if the increase of AMR is not controlled [1,2]. Currently, many pathogenic bacteria are highly prevalent on antimicrobial resistance in many countries. Clinical doctors are frequently forced to use second-line or last-line antibiotics for bacterial infections. Consequently, more multidrug- or even pandrug-resistant bacteria have emerged [3]. Carbapenem resistance is a particularly critical case. The World Health Organization has listed carbapenem-resistant Gram-negative bacilli including *Acinetobacter baumannii*, *Pseudomonas aeruginosa,* and Enterobacteriaceae as critical pathogens for which discovery and development of new antibiotics are of urgent priority due to limited treatment options available for infection by these bacteria [4].

*A. baumannii* is a predominant pathogen associated with nosocomial and community infections at various body sites including the bloodstream, respiratory tract, urinary tract, surgical sites, and wounds [5]. Due to the difficulty in species identification, three clinically relevant species, *A. baumannii*, *Acinetobacter nosocomialis,* and *Acinetobacter pittii,* are grouped with an environmental species, *Acinetobacter calcoaceticus,* into the *A. calcoaceticus*–*A. baumannii* (Acb) complex [6]. This group of bacteria pose a high challenge in clinical settings because of their high AMR [6]. The development of multidrug resistance (MDR) in *A. baumannii* is mediated through genomic mutations or acquired antimicrobial resistance genes. Carbapenem treatment for MDR *A. baumannii* infections leads to an increase in the prevalence of carbapenem-resistant *A. baumannii* (CRAB). A longitudinal surveillance program in Taiwan showed an increase of CRAB prevalence from 3.4% in 2002 to 58.7% in 2012 [7]. The frequency of CRAB was approximately 70% in the Acb complex isolated from hospitals in Taiwan [8]. A 2019 report from the Centers for Disease Control in the United States listed CRAB as an urgent threat to public health [9]. Because of the significant increase in CRAB, colistin has been used to treat CRAB infections despite its nephrotoxicity. Eventually and not unexpectedly, the emergence of colistin- and carbapenem-resistant *A. baumannii* (CCR-AB) was reported [10,11,12].

Colistin is a cationic lipopeptide that interacts directly with lipid A of lipopolysaccharide (LPS). Insertion of colistin inside the outer membrane of Gram-negative bacteria results in membrane disruption and cell death [13]. Mechanisms of colistin resistance in *A. baumannii* are mainly caused by chemical modifications of LPS mediated through dysregulation or acquisition of LPS modifying enzymes [14,15,16]. Mutations in the two-component system, *pmrA* (response regulator) and *pmrB* (kinase sensor), result in upregulation of the downstream gene *pmrC,* which encodes a lipid A phosphoethanolamine (pEtN) transferase. Overproduction of PmrC increases the addition of pEtN to lipid A, lowering its affinity to colistin [17,18,19,20]. Overexpression of other LPS modifying enzymes, such as EptA (PmrC homolog) and NaxD (acetyl-galactosamine deacetylase), which are under *pmrAB* regulation, has also been reported to confer colistin resistance [19,21]. Recently, plasmid-borne pEtN transferases, Mcr-1 and Mcr-4.3, were found in *A. baumannii* [14,15]. Mutations in LPS biosynthesis genes, such as *lpxA*, *lpxC,* and *lpxD* (lipid A) [22,23] or *lpsB* or *lptD* (non-lipid A) [24,25], may also confer colistin resistance. Other colistin resistance mechanisms include reduction of biosynthesis of osmoprotective amino acids or expression of efflux pumps [24,26,27].

Alteration of LPS structure may change the fitness of the CCR-AB cultures and their virulence. Impaired virulence and in vivo fitness were observed in a laboratory-evolved colistin-resistant strain of *A. baumannii* with a *pmrB* mutation [28] or in a clinical isolate from a patient without signs of infection [29]. A strong cost of fitness and virulence was found in colistin resistance with LPS loss rather than with LPS modification in *A. baumannii* [30,31]. In contrast, there have been reports showing no reduction in the growth or virulence in colistin-resistant *A. baumannii* [32,33].

A meta-analysis showed that the highest prevalence of colistin resistance in *A. baumannii* was in Lebanon (17.5%) followed by China (12%) among 41 countries surveyed [34]. The increasing trend of colistin resistance from 2000 to 2017 was higher in South-East Asia and the Eastern Mediterranean than in Europe and Africa [34]. The prevalence of colistin-resistant *A. baumannii* in Taiwan was 2.5% according to a meta-analysis spanning from 2013 to 2016 [34]. Different collections from Taiwan showed various degrees of colistin-resistant *A. baumannii*. A national surveillance from Intensive Care Units in Taiwan reported the prevalence as 6% in 2005 and 10.1% in 2016 [35,36]. Another study in 2007 showed the prevalence was 10.4% across Eastern, Southern, and Northern Taiwan [37]. None of these reports have explored the molecular characteristics and resistance mechanisms of the colistin-resistant *A. baumannii* isolates.

In this study, we collected CRAB from a teaching hospital in Taiwan from 2017 to 2018. The prevalence of colistin resistance was 1.5% among the CRAB isolates. Seven CCR-AB isolates were identified and chosen for characterization at the molecular level including multilocus sequence types, capsule types, and resistome profiles. In addition, mechanisms of colistin resistance were investigated. Heteroresistance to colistin was found in one of the seven isolates. Virulence-associated phenotypes such as growth, biofilm formation, and virulence in a wax moth infection model were studied. 

## 2. Materials and Methods

### 2.1. Collection of Clinical Isolates, Species Identification, and Antimicrobial Susceptibility Testing

Carbapenem-resistant Acb isolates were collected at Taipei Medical University Hospital from 2017 to 2018. From them, seven isolates resistant to colistin and carbapenem were further characterized. Antimicrobial susceptibility testing (AST) was performed using BD Phoenix^TM^ Automated Identification and Susceptibility Testing System (BD Diagnostics System, Sparks, MD, USA). Validation of colistin resistance was performed using the broth microdilution method in cation-adjusted Mueller–Hinton broth. Interpretation of AST was based on the criteria of Clinical & Laboratory Standards Institute (CLSI) guideline 2018 [38]. Species identification was based on *gyrB* multiplex PCR [39] supplemented by *rpoB* PCR and sequencing. Detection of oxacillinase genes including *bla*_OXA-51_, *bla*_OXA-23_, *bla*_OXA-24_, and *bla*_OXA-58_ was performed using PCR. The PCR primers are listed in Table S1.

### 2.2. Whole-Genome Sequencing and Molecular Characterization

Genomic DNA was extracted from overnight cultures in tryptic soy broth at 37 °C using a Wizard Genomic DNA Purification Kit (Promega, Madison, WI, USA). Whole-genome sequencing was performed using the Illumina platform with a read length of 150 bp in pairs. The depth of each isolate was over 30. The obtained short reads of each isolate were assembled de novo using CLC Genome Workbench (QIAGEN, Hilden, Germany). The numbers of contigs for each isolate ranged from 187 to 494. The assembled draft genomes were used to determine molecular features. Two schemes of MLSTs, Pasteur [40] and Oxford [41], were analyzed using MLST 2.0 (https://cge.cbs.dtu.dk/services/MLST/, accessed on 21 July 2020). Capsule types were determined using Kaptive [42]. Acquired antimicrobial resistance genes were detected with coverage >60% and identity >90% using ResFinder 3.2 [43]. Draft genomes of these isolates have been deposited at the National Center for Biotechnology Information with the following accession numbers: JAENTF000000000 (T1060317), JAENTE000000000 (T1060361), JAENTD000000000 (T1060578), JAENTC000000000 (T1060580), JAENTB000000000 (T1070171), JAENTA000000000 (T1070213), and JAENSZ000000000 (T1070678).

### 2.3. Sequence Analysis of Genes Related to Colistin Resistance and Virulence

Sequences of genes related to virulence and colistin resistance in each isolate were compared pairwise with the reference strain (*A. baumannii* ACICU). Common genes related to colistin resistance including *pmrCAB* operon, *lpxA*, *lpxC,* and *lpxD* were analyzed by BLAST and validated by PCR sequencing (primers listed in Table S1). Other reported genes (*pheS, pldA*, *vacJ*, *lpsB,* and *miaA*) were also examined by sequence comparison with the reference strain. Virulence genes were detected on the Virulence Factors Database (VFDB) server [44]. The criteria of BLASTN analysis were score >100 and identity >90%.

### 2.4. Detection of Gene Expression Levels

The expression levels of *pmrC*, *eptA*, and *naxD* were determined using reverse transcriptase quantitative PCR. Bacterial cultures in exponential phase (OD_600_ of 0.5~0.7) were harvested and treated with RNA*later*^TM^ solution (Thermo Fisher Scientific, Carlsbad, CA, USA) for one hour at room temperature. Total RNA was extracted using PureLink^TM^ RNA Mini Kit (Thermo Fisher Scientific, Carlsbad, CA, USA). DNA was eliminated by DNase I (Lucigen Corporation, Middleton, WI, USA) treatment. RNA was further purified using RNA Clean and Concentrator^TM^-5 (Zymo Research, Irvine, CA, USA). Concentration of total RNA was measured using NanoDrop^®^ ND-1000 spectrophotometer (Thermo Fisher Scientific, Waltham, MA, USA) and integrity of RNA was evaluated using agarose gel electrophoresis. Complementary DNA was synthesized using High-capacity cDNA Reverse Transcription Kits (Applied Biosystems, Waltham, MA, USA). Three targeted genes (*eptA*, *naxD,* and *pmrC*) and the reference gene (*rpoB*) were quantified using SensiFAST^TM^ SYBR^®^ Hi-ROX Kit (Meridian Bioscience, Cincinnati, OH, USA) with respective primer pairs (listed in Table S1) in Applied Biosystems 7300 Real-Time PCR System (Thermo Fisher Scientific, Waltham, MA, USA). Relative expression levels were determined using the 2^-∆∆Ct^ method [45]. *A. baumannii* ATCC 19606 was chosen as the reference. Statistical significances between the CCR-AB isolates and the reference were calculated using one-way ANOVA. Statistical analyses and graphs were produced using GraphPad Prism 5.

### 2.5. Lipopolysaccharide Analysis by SDS-PAGE

LPS extraction from overnight bacterial cultures on LB agar was performed using an LPS Extraction Kit (Abcam, Cambridge, UK). The extracted LPS was mixed in 2X SDS loading buffer, boiled for 15 min, and subjected to SDS-PAGE in 15% acrylamide gels. After electrophoresis, the gel was stained with silver stain.

### 2.6. Measurement of Generation Time

An overnight bacterial culture in Mueller–Hinton broth (MHB) was harvested and adjusted to OD_600_ 0.1. The 250 μL of diluted suspension was inoculated into 50 mL MHB in a 250-mL flask and shaken at 150 r.p.m. at 37 °C. OD_600_ value was measured every 30 min until the end of the exponential phase. Log phase determinations were used to calculate the generation time [46].

### 2.7. Biofilm Formation Ability

Biofilm formation was assessed either by static culture in a 96-well polystyrene microplate or aerated culture with a plate covered by a lid with pegs. Biofilm was measured using the crystal violet method [47]. For measurement of biofilm production in static cultures, each isolate was cultured in 3 mL MHB for 16 h at 37 °C. The cultures were adjusted to OD_600_ of 0.01, and 100 µL of the dilution was transferred into a 96-well polystyrene microplate (eight replicate wells per isolate) and incubated at 37 °C for 24 h without shaking. After removal of the bacterial cultures, the wells were washed with sterile water, stained with 150 µL of 0.1% (*w/v*) crystal violet at room temperature for 30 min, washed three times with water, and then air-dried. For measurement of biofilm formation in aerated cultures, 200 µL of diluted suspension was aliquoted into an MBEC assay^®^ biofilm inoculator with a 96-well base (Innovotech, Edmonton, Canada) and shaken at 110 r.p.m. for 16 h in a 37 °C incubator. Biofilm formed on the pegs was rinsed with water three times, stained in 200 µL of 0.1% (*w/v*) crystal violet at room temperature for 30 min, washed with water, and dried. The stained biofilm was dissolved in 33% acetic acid solution and absorbance at 550 nm was measured in a spectrophotometer. ATCC 19606 served as a positive control and MHB blank as a negative control. Statistical significances were determined by one-way ANOVA. Statistical analyses and graphs were constructed using GraphPad Prime 5.

### 2.8. Determination of Colistin Heteroresistance

Bacterial isolates were cultured in 3 mL LB broth in an orbital shaker at 150 r.p.m. at 37 °C for 16–20 h. The overnight cultures were serially diluted from 10^1^ to 10^7^ folds. The diluted suspensions were spread onto LB agar plates containing colistin in concentrations ranging from 0 to 128 mg/L in 2-fold increments and incubated at 37 °C for 16 h. Colony formation units (CFU) on the agar plates were counted. The fraction of colistin-resistant bacteria at each concentration was determined by dividing them into CFU on colistin-free agars. Isolates that exhibited a resistance fraction between 10^−7^ and 5 × 10^−1^ at 16 mg/L of colistin were designated heteroresistant [48]. 

### 2.9. Virulence Analysis in Galleria Mellonella Infection Model

A 1.5 mL aliquot of overnight culture in LB was centrifuged and the pellet was resuspended into 500 μL PBS. The suspension was adjusted to OD_600_ of 0.01 (approximately 10^7^ CFU/mL). Late-stage larvae of wax moth *Galleria mellonella* reared in-house at 28 °C were harvested. Fifteen larvae weighing 250–350 mg were injected at the last left proleg with 10 μL of the bacterial suspension using a needle syringe [49]. Survival of the larvae was scored after 24, 48, and 72 h. A reference strain, *A. baumannii* AYE, and PBS solution were used as positive and negative controls, respectively. The Kaplan–Meier survival curve and statistical analysis were constructed using GraphPad Prism 5.

## 3. Results and Discussion

### 3.1. Epidemiology of Colistin- and Carbapenem-Resistant A. baumannii (CCR-AB)

Seven of our 452 CRAB isolates from 2017 to 2018 collected from sputum specimens of five hospitalized patients were resistant to colistin. The CCR-AB prevalence (1.5%) was significantly lower than that previously reported in Taiwan (10.4% in 2007 [37] and 10.1% in 2016 [36]) and in Greece (32.8% in 2015–2017) [50]. The antimicrobial susceptibility of the seven isolates is shown in Table 1. While one isolate (T1060587) exhibited multidrug resistance (MDR), the remaining six were extensively drug-resistant (XDR). All were sensitive to minocycline and resistant to colistin, imipenem, meropenem, gentamicin, and ciprofloxacin. Minimal inhibitory concentration of colistin ranging from 0.5 to 16 mg/L was reported for previous isolates in Taiwan [36]. However, high resistance to colistin, i.e., at least an eight-fold increase in the breakpoint, was observed in our isolates.

### 3.2. Molecular Characterizations of CCR-AB Isolates

Draft genomes of the seven isolates were determined from which molecular typing and resistome analysis were performed (Table 2). Three groups may be classified based on their molecular characteristics. Sequence types (STs) of the six XDR isolates were ST2 (Pasteur) or alternatively global clone 2 (GC2), with KL2 capsule type. Among them, two isolates (T1060317 and T1060361) from the same patient were ST544 and unknown ST, and the other four were ST208 and ST1806 due to the presence of two *gdhB* alleles in the Oxford MLST scheme. The MDR isolate was ST136 (Pasteur) with KL107 capsule type, but a novel sequence type was identified near ST460 or ST1092 in the Oxford MLST scheme. 

Acquired resistome analysis showed the presence of OXA-23 oxacillinase hydrolyzing carbapenems in all seven isolates. In addition, all six GC2 isolates except T1070213 harbored nearly identical antimicrobial resistance genes (ARGs) for β-lactams, aminoglycosides, macrolides, phenicols, sulfonamides, and tetracyclines. T1070213 lacked the ARGs for macrolides and phenicols. However, the ST136 (Pasteur) isolate only contained ARGs for β-lactams and aminoglycosides. This finding was consistent with its MDR phenotype. In addition, this MDR isolate possesses three β-lactamases genes including a *bla*_OXA-51_ variant (*bla*_OXA-317_), *bla*_OXA-23_, and *bla*_ADC-25_ but not *bla*_TEM-1D_.

### 3.3. Mutation Analysis of Genes Conferring Colistin Resistance

**Figure 1:**
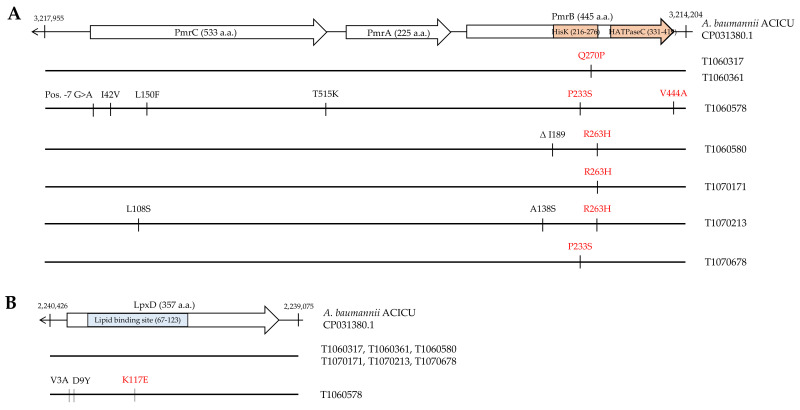
Amino acid substitutions in PmrC, PmrA, PmrB (**A**), and LpxD (**B**) in the CCR-AB isolates. The genetic map of the reference strain, ACICU, is displayed on the top with nucleotide numbers indicated. The open arrows depict the relevant genes with the names and size (in amino acid) shown. The functional domains in PmrB and LpxD are colored in orange and blue, respectively. Amino acid substitutions in the CCR-AB isolates are pictured below. Previously reported mutations are marked in red. Newly identified mutations are marked in black.

The PmrAB two-component system mediates colistin resistance in many Gram-negative bacteria including *A. baumannii* [51]. It regulates several genes encoding LPS modifying enzymes including *pmrC*, *naxD,* and *eptA*. Non-synonymous mutations in *pmrAB* may dysregulate its downstream genes. Compared to the reference strain, the seven isolates harbored a number of non-synonymous mutations in the histidine kinase domain of *pmrB* (Figure 1A). Three of the resulted amino acid substitutions (P233S, R263H, and Q270P) in PmrB are known to confer colistin resistance [17,52,53]. Two isolates, T1060578 and T1070213, harbored additional new substitutions outside the histidine kinase domain of PmrB, the effects of which are not known. No mutation was found in *pmrA* in the seven isolates. New mutations in *pmrC* were found in two isolates, where T1060578 (ST136, Pasteur) harbored three non-synonymous mutations in *pmrC* and one mutation in its promoter region. An L108S substitution in PmrC was found in T1070213. Pre-existing mutations in the population or mutations generated from antibiotic-induced stress responses are known to be selected under antibiotic treatments [54,55]. Indeed, our five patients, from whom the seven isolates were collected, had previously received colistin treatment.

Mutations in LPS biosynthesis genes such as *lpxA*, *lpxC,* and *lpxD* may also result in colistin resistance [22,23]. Six of the seven isolates did not possess any mutation in these three genes, while T1060578 contained three non-synonymous mutations in *lpxD*. One of the substitutions (K117E) is at the lipid binding site, which has been associated with colistin resistance (Figure 1B). No mutation was found in other genes associated with colistin resistance including *pldA*, *pheS*, *vacJ*, *lpsB,* and *miaA*. These findings are consistent with previous reports showing that mutations in the histidine kinase domain of *pmrB* were mainly responsible for colistin resistance in clinical *A. baumannii* isolates [17,18,19].

### 3.4. Effects of PmrB and LpxD Mutations

**Figure 2:**
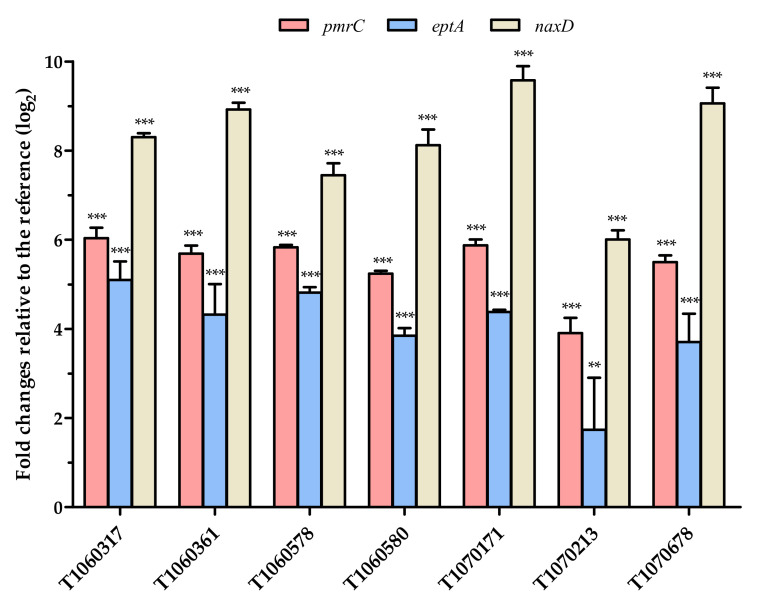
Gene expression levels of LPS modifying enzymes in the CCR-AB isolates. Expression of *pmrC*, *eptA,* and *naxD* was compared to that of the reference strain ATCC 19606. The result was expressed in folds of changes in log_2_ scale. *rpoB* was used as an internal reference for each isolate. The error bars indicate standard errors from three replicate determinations. Statistical analysis was conducted by one-way ANOVA with Dunnett’s test for comparison of individual isolate and the reference. **, 0.001 < *p* ≤ 0.01; ***, *p* ≤ 0.001.

Activation mutations in *pmrB* (histidine kinase) result in constitutive expression of its downstream genes through the action of *pmrA* (response regulator). Two of the *pmrB*-regulated genes, *pmrC* and *eptA*, encode phosphoethanolamine transferases, which add phosphoethanolamine (pEtN) to neutralize the negative charge on lipid A. Another *pmrB*-regulated gene, *naxD,* encodes an acetyl-galactosamine deacetylase, which modifies lipid A with galactosamine. Expression levels of these three genes in the seven isolates (Figure 2) were significantly increased compared to those in the reference culture. In each isolate, the expression of *naxD* was highest, followed by that of *pmrC* and *eptA*. T1070213, which was highly resistant to colistin, exhibited lower expression of these three genes than the other isolates with similar or lower colistin resistance. Comparison of T1070171 and T1070213 (from the same patient) showed an additional A138S substitution in PmrB in T1070213. It is possible that A138S attenuates the kinase activity of PmrB, resulting in reduced expression of the downstream genes.

**Figure 3:**
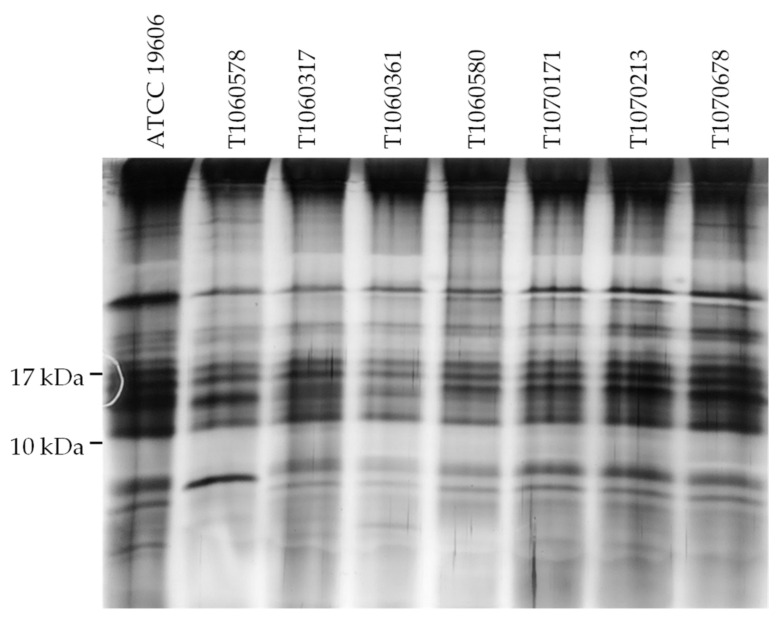
LPS profiles of the CCR-AB isolates. LPS extracted from each isolate was electrophoresed in 15% polyacrylamide gel and silver stained. The sizes of protein markers are shown to the left. The reference strain, ATCC 19606, represents a wild-type profile for comparison.

Mutations in *lpxA*, *lpxC,* or *lpxD* result in deficiency of LPS biosynthesis. The LPS profiling of the seven isolates was analyzed to evaluate the effect of mutations in LPS synthesis enzymes (Figure 3). Only T1060578 isolate with a K117E substitution in LpxD displayed a different LPS pattern compared to the reference and other CCR-AB isolates, all of which do not have an LpxD mutation. A single strong band near the bottom of the analyzed gel was observed in T1060578 but not the others.

These results indicate that colistin resistance in the CCR-AB isolates may involve either modifications of LPS or alterations in LPS biosynthesis. In particular, T1060578 appears to possess both mechanisms.

### 3.5. Fitness, Biofilm Formation, and Heteroresistance to Colistin

**Figure 4:**
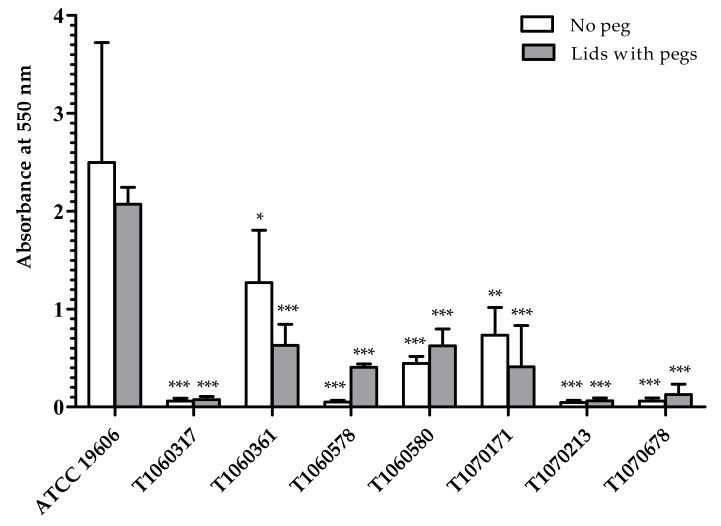
Biofilm formation abilities of the CCR-AB isolates. Individual bacterial suspension was cultured overnight in conventional 96-well plates (‘No peg’; open bars) or in 96-wells with lid pegs (filled bar). Biofilms were measured by crystal violet staining. The ordinate represents measured absorbance at 550 nm. The error bars indicate standard errors from three replicate determinations. Statistical analysis was calculated using one-way ANOVA with Dunnett’s test for comparison to the reference, ATCC 19606. Numbers of asterisks indicate significant difference in different levels with *p* ≤ 0.001 (***), 0.001 < *p* ≤ 0.01 (**), and 0.01 < *p* ≤ 0.05 (*).

Alterations in LPS biosynthesis may affect bacterial growth and biofilm formation [47,56]. To understand the possible effects of activated *pmrB* on the growth of the seven isolates, generation time was determined for each isolate. No significant difference in generation time was observed among them and the reference (Table S2), indicating no fitness cost was exerted on these isolates. Biofilm formation under aerated or non-aerated conditions was lower in the isolates than that in the reference (Figure 4): not detectable in the three GC2 isolates (T1060317, T1070213, and T1070678) and reduced formation in the remaining four. Interestingly, members of the two pairs of isolates (T1060317/T1060316, T1070171/T1070213) each from the same patient, differed significantly in biofilm production despite their highly similar genotypes.

Deficiency in biofilm formation has been reported in clinically isolated colistin-resistant *A. baumannii*, which either contained mutations in *lpx* alone or mutations in *pmrB* and other biofilm-associated genes [57]. In silico analysis of biofilm-associated genes showed partial or complete deletion of *csu* fimbrial operon, a known biofilm-associated gene cluster in two of the three isolates (T1070213 and T1070678) deficient in biofilm formation (Table 3).

**Figure 5:**
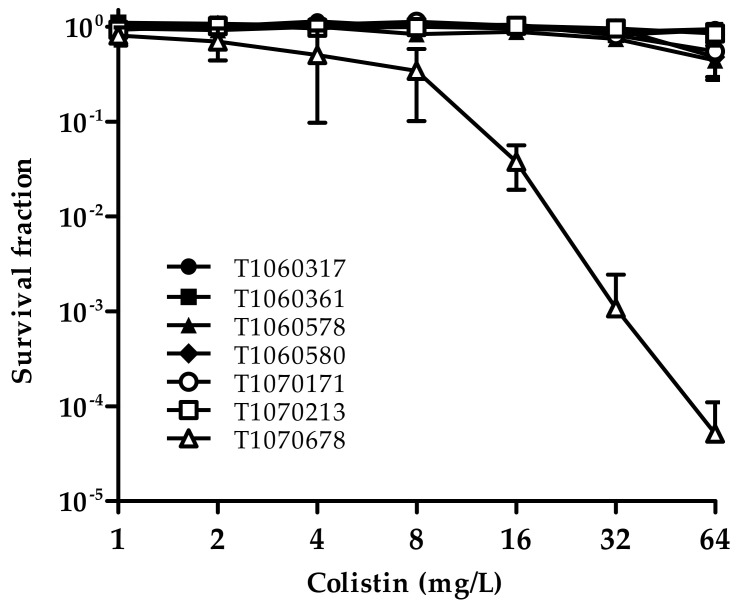
Population analysis profiles of the CCR-AB isolates. An overnight culture of each isolate was serial diluted 10-fold. The diluted bacterial suspension was plated onto LB agar, with colistin concentration ranging from 0 to 64 mg/L. CFU was counted in each agar. The survival fraction in each colistin concentration was calculated by comparing it to CFU on colistin-free medium. The results present the average of three replicates with standard errors depicted by the error bars.

A 33% prevalence of colistin heteroresistance in *Acinetobacter* spp. based on a meta-analysis has been reported [58]. In the seven CCR-AB isolates, six displayed stable resistance to colistin with a higher than 40% survival rate at the highest colistin concentration tested (64 mg/L). The other isolate, T1070678, displayed heteroresistance with 3.8 × 10^−2^ survival at 16 mg/L of colistin (Figure 5). T1070678 contains a P233S substitution in PmrB, which is known to be associated with stable resistance [59]. No reported mutations in *pmrA* or *pmrB* are known to be involved in heteroresistance. It is possible that colistin heteroresistance in T1070678 may be attributed to elevated efflux pumps, as has been previously reported [60].

### 3.6. Virulence to Wax Moth

**Figure 6:**
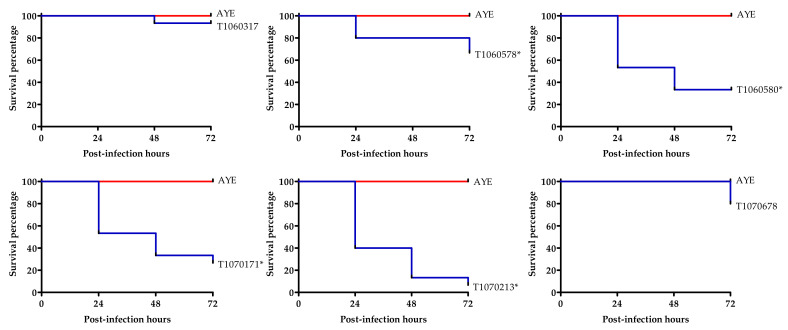
Survival curve of wax moth larvae after injection of the CCR-AB isolates. Fifteen larvae at the late larva stage were injected with 10^5^ CFU of each isolate culture. Survival of the injected larvae after 24, 48, and 72 h were scored. The survival curves were plotted using Kaplan–Meier analysis. PBS solution injection (as a negative control) exhibited no lethality during the observation period. *A. baumannii* AYE was used as a reference for comparison. Representative results from three independent replicates are shown. The asterisks (*) indicate the significant difference (*p* < 0.01) between the reference and tested isolate.

Colistin resistance in *A. baumannii* isolates has been shown to be associated with virulence reduction [30,61]. Virulence of the CCR-AB isolates was analyzed using the wax moth infection model. Most of our CCR-AB isolates were more virulent to different degrees than the reference strain *A. baumannii* AYE (Figure 6). Three (T1060580, T1070171, and T1070213) out of four isolates classified as ST208 and ST1806 (Oxford) killed more than 60% of larvae 72 h post-injection. The most virulent isolate, T1070213, killed more than 80% of larvae 48 h post-injection. Such virulence of T1070213 is comparable to that of a known virulent strain AB5057 using the same test model [62]. Interestingly, T1070213, isolated from the same patient as T1070171 but two weeks later, exhibited significantly higher virulence than the latter. The high virulence of T1070213 is of interest. While hypervirulent CRAB isolates outside of ST208 have been described [63], no hypervirulent ST208 isolate has been reported. It is possible that the high virulence of T1070213 is due to higher resistance to the complement system in the innate immunity in wax moth.

### 3.7. In Silico Analysis of Virulence Factors

In silico analysis of 39 known virulence factors from the draft genomes shows high similarities (Table 3). Identities and coverage in these virulence factors were nearly the same among the GC2 isolates, although the ST208 and ST1806 (Oxford) isolates were more virulent to moth larvae than the ST544 (Oxford) isolates. It implies that other factors contribute to the higher virulence of the ST208 and ST1806 (Oxford) isolates. One isolate (T1070678) in ST208 and ST1806 (Oxford) has lost the *csu* fimbriae operon and part of the acinetobactin biosynthesis gene cluster and exhibited the avirulent phenotype, suggesting that the missing genes are required for virulence. Interestingly, T1070213, despite lacking complete *csu* operon (encoding a virulent factor), is more virulent than its paired isolate, T1070171. We suspect that T1070213 may contain one or more novel virulence factors.

### 3.8. Potentially Alternative Therapies against MDR Bacteria

The need to discover new approaches against MDR bacteria is urgent. Combination therapies with compounds to increase membrane permeability or to reduce efflux pump activity have been proposed [64]. The use of efflux pump inhibitors as antibiotic adjuvants may elevate the efficacy of antibiotics. Many natural and synthetic compounds acting as efflux pump inhibitors have been found to be effective against Gram-positive and Gram-negative MDR bacteria [65,66]. Some plant products such as essential oils have shown broad-spectrum activities against bacteria and fungi [67,68]. In particular, oregano essential oil has been shown to inhibit the growth of MDR *A. baumannii* and function synergistically with polymyxin B [69]. Another essential oil from *Zingiber cassumunar* Roxb exhibited antibacterial activities and enhancing effects for several classes of antibiotics against *A. baumannii,* including XDR isolates [70]. Alternative therapies such as these may aid our continuing fight against MDR bacteria.

## 4. Conclusions

In this study, we collected seven CCR-AB isolates from a teaching hospital in Taiwan and performed genomic epidemiology to uncover their molecular features and resistome profiles. Most of the isolates were endemic GC2 clones and acquired multiple antimicrobial resistance determinants. Carbapenemase OXA-23 is responsible for carbapenem resistance, while colistin resistance was mediated through *pmrB* mutations that alter lipid A and/or LPS in our isolates. Colistin heteroresistance was found in one isolate. Different biofilm formation abilities and virulence were found among GC2 isolates and between paired isolates from the same patients, implying other genetic traits may be involved in these phenomena.

## Figures and Tables

**Table 1:** Antimicrobial susceptibility testing of the CCR-AB isolates.

Isolate	Isolation Date	Patient ID	Minimal Inhibition Concentration (mg/L) ^1^														
			IMP	MEM	CTX	CAZ	CRO	FEP	SAM	TZP	GEN	AMK	CIP	LVX	SXT	COL ^2^	MIN
T1060317	11 July 2017	PT0044	>4 (R)	>4 (R)	>32 (R)	>16 (R)	>32 (R)	>16 (R)	>16/8 (R)	>64/4 (R)	>8 (R)	>32 (R)	>2 (R)	>4 (R)	>2/38 (R)	>128 (R)	4 (S)
T1060361	25 July 2017	PT0044	>4 (R)	>4 (R)	>32 (R)	>16 (R)	>32 (R)	>16 (R)	>16/8 (R)	>64/4 (R)	>8 (R)	>32 (R)	>2 (R)	>4 (R)	>2/38 (R)	128 (R)	4 (S)
T1060578	10 November 2017	PT0345	>4 (R)	>4 (R)	8 (S)	2 (S)	4 (S)	8 (S)	8/4 (S)	64/4 (I)	>8 (R)	>32 (R)	>2 (R)	4 (I)	≤0.5/9.5 (S)	64 (R)	4 (S)
T1060580	13 November 2017	PT0348	>4 (R)	>4 (R)	>32 (R)	16 (I)	>32 (R)	16 (I)	8/4 (S)	64/4 (I)	>8 (R)	>32 (R)	>2 (R)	>4 (R)	>2/38 (R)	>128 (R)	4 (S)
T1070171	13 March 2018	PT0512	>4 (R)	>4 (R)	>32 (R)	>16 (R)	>32 (R)	>16 (R)	>16/8 (R)	>64/4 (R)	>8 (R)	>32 (R)	>2 (R)	>4 (R)	>2/38 (R)	>128 (R)	4 (S)
T1070213	30 March 2018	PT0512	>4 (R)	>4 (R)	>32 (R)	>16 (R)	>32 (R)	>16 (R)	>16/8 (R)	>64/4 (R)	>8 (R)	≤ 8 (S)	>2 (R)	>4 (R)	>2/38 (R)	>128 (R)	4 (S)
T1070678	23 October 2018	PT0712	>4 (R)	>4 (R)	>32 (R)	>16 (R)	>32 (R)	>16 (R)	8/4 (S)	>64/4 (R)	>8 (R)	>32 (R)	>2 (R)	>4 (R)	>2/38 (R)	64 (R)	4 (S)

^1^ Abbreviations of tested antibiotics: IMP, imipenem; MEM, meropenem; CTX, cefotaxime; CAZ, ceftazidime; CRO, ceftriaxone; FEP, cefepime; SAM, ampicillin-sulbactam; TZP, piperacillin-tazobactam; GEN, gentamicin; AMK, amikacin; CIP, ciprofloxacin; LVX, levofloxacin; SXT, trimethoprim-sulfamethoxazole; COL, colistin; MIN, minocycline. ^2^ Minimal inhibition concentration of colistin was confirmed using the broth microdilution method.

**Table 2:** Molecular characterizations and acquired antimicrobial resistance genes in the CCR-AB isolates.

Isolate	Patient ID	ST ^1^ (Pasteur/Oxford)	Capsule Type	Acquired Resistance Genes					
				β-lactams	Aminoglycosides	Macrolides	Phenicols	Sulfonamides	Tetracyclines
T1060317	PT0044	2/544 and unknown	KL2	*bla*_ADC-25_, *bla*_OXA-23_, *bla*_OXA-66_, *bla*_TEM-1D_	*aac(6’)-Ib3, aadA1, aph(3”)-Ib, aph(3’)-Ia, aph(6’)-Id, armA*	*mph(E), msr(E)*	*catB8*	*sul1, sul2*	*tet(B)*
T1060361	PT0044	2/544 and unknown	KL2	*bla*_ADC-25_, *bla*_OXA-23_, *bla*_OXA-66_, *bla*_TEM-1D_	*aac(6’)-Ib3, aadA1, aph(3”)-Ib, aph(3’)-Ia, aph(6’)-Id, armA*	*mph(E), msr(E)*	*catB8*	*sul1, sul2*	*tet(B)*
T1060578	PT0345	136/~460 or 1092 ^2^	KL107	*bla*_ADC-25_, *bla*_OXA-23_, *bla*_OXA-317_	*ant(2”)-Ia, aph(3’)-VI*	-	-	-	-
T1060580	PT0348	2/208 and 1806	KL2	*bla*_ADC-25_, *bla*_OXA-23_, *bla*_OXA-66_, *bla*_TEM-1D_	*aac(6’)-Ib3, aadA1, aph(3”)-Ib, aph(3’)-Ia, aph(6’)-Id, armA, ant(2”)-Ia, aph(3’)-VI*	*mph(E), msr(E)*	*catB8*	*sul1, sul2*	*tet(B)*
T1070171	PT0512	2/208 and 1806	KL2	*bla*_ADC-25_, *bla*_OXA-23_, *bla*_OXA-66_, *bla*_TEM-1D_	*aac(6’)-Ib3, aadA1, aph(3”)-Ib, aph(3’)-Ia, aph(6’)-Id, armA*	*mph(E), msr(E)*	*catB8*	*sul1, sul2*	*tet(B)*
T1070213	PT0512	2/208 and 1806	KL2	*bla*_ADC-25_, *bla*_OXA-23_, *bla*_OXA-66_, *bla*_TEM-1D_	*aph(3”)-Ib, aph(6’)-Id ant(2”)-Ia, aph(3’)-VI,*	-	-	*sul2*	*tet(B)*
T1070678	PT0712	2/208 and 1806	KL2	*bla*_ADC-25_, *bla*_OXA-23_, *bla*_OXA-66_, *bla*_TEM-1D_	*aac(6’)-Ib3, aadA1, aph(3”)-Ib, aph(3’)-Ia, aph(6’)-Id, armA*	*mph(E), msr(E)*	*catB8*	*sul1, sul2*	*tet(B)*

^1^ ST: Sequence types from both Pasteur and Oxford schemes of multilocus sequence types in *A. baumannii*. ^2^ The symbol “~” means the nearest ST.

**Table 3:** In silico detection of virulence factors in the CCR-AB isolates.

Category	Gene	Product	ST2/ST544		ST2/ST208&1806				ST136
			T1060317	T1060361	T1060580	T1070171	T1070213	T1070678	T1060578
Adherence	*ompA*	Outer membrane protein OmpA	100/100 ^1^	100/100	100/100	100/100	100/100	100/100	98/100
Quorum sensing	*abaR*	DNA-binding HTH domain-containing protein	98/100	98/100	98/100	98/100	98/100	98/100	98/100
	*abaI*	N-acyl-L-homoserine lactone synthetase	98/100	98/100	98/100	98/100	98/100	98/100	98/100
Enzymes	*plcD*	Phospholipase	99/99.8	99/99.8	98/99.8	99/99.8	99/99.8	99/99.8	95/99.8
	*plc*	Phospholipase C	100/100	100/100	100/100	100/100	100/100	100/100	97/100
	*plc*	Phospholipase C	99/100	99/100	99/100	99/100	99/100	99/100	99/100
Biofilm -Csu fimbriae	*csuC*	Csu pilus chaperone protein CsuC	100/100	100/100	100/100	100/100	100/100	Not found	98/100
	*csuA/B*	Csu pilus major pilin subunit CsuA/B	100/100	100/100	100/100	100/100	100/100	Not found	99/100
	*csuB*	Csu pilus subunit CsuB	100/100	100/100	100/100	100/100	100/100	Not found	98/100
	*csuD*	Csu pilus subunit CsuD	99/100	99/100	99/100	99/100	100/20.8	Not found	98/100
	*csuA*	Csu pilus usher protein CsuA	100/100	100/100	100/100	100/100	100/100	Not found	98/100
	*csuE*	Csu pilus tip adhesin CsuE	99/100	99/100	99/100	99/97.9	Not found	Not found	95/100
Ade efflux pumps	*adeG*	Cation/multidrug efflux pump AdeG	100/100	100/100	100/100	100/100	100/100	100/100	95/100
	*adeH*	Outer membrane protein AdeH	100/100	100/100	100/100	100/100	100/100	100/100	98/100
	*adeF*	Membrane-fusion protein AdeF	100/100	100/100	100/100	100/100	100/100	100/100	98/100
Biofilm-PAGN ^2^	*pgaB*	PNAG N-deacetylase PgaB	99/100	99/100	99/100	99/100	99/100	99/100	97/100
	*pgaA*	PNAG export porin PgaA	98/99.8	98/99.8	98/99.8	98/99.8	98/99.8	98/99.8	97/100
	*pgaC*	PNAG synthase	100/100	100/100	100/100	100/100	100/100	100/100	97/100
	*pgaD*	PNAG biosynthesis protein PgaD	100/100	100/100	100/100	100/100	100/100	100/100	97/100
Regulation	*bfmS*	Signal transduction histidine kinase BfmS	100/100	100/100	100/100	100/100	100/100	100/100	98/100
	*bfmR*	Biofilm-controlling response regulator BfmR	100/100	100/100	100/100	100/100	100/100	100/100	99/100
Iron uptake	*barA*	Siderophore efflux system of the ABC superfamily BarA	100/100	100/100	100/100	100/100	100/100	Not found	97/100
	*barB*	Siderophore efflux system of the ABC superfamily BarB	99/99.9	99/99.9	99/99.9	99/99.9	99/99.9	Not found	97/99.9
	*basA*	Acinetobactin biosynthesis protein BarA	100/100	100/100	100/100	100/100	100/100	100/100	96/100
	*basB*	Non-ribosomal peptide synthetase BarB	99/100	99/100	99/100	99/100	99/100	99/100	96/100
	*basC*	Acinetobactin biosynthesis protein BasC	100/99.2	100/99.2	100/99.2	100/99.2	100/99.2	100/99.2	97/99.2
	*basD*	Acinetobactin biosynthesis protein BasD	99/100	99/100	99/100	99/100	99/100	99/100	96/100
	*basF*	Aryl carrier protein BasF	100/100	100/100	100/100	100/100	100/100	100/100	97/100
	*basG*	Acinetobactin biosynthesis protein BasF	100/100	100/100	100/100	100/100	100/100	Not found	97/100
	*basH*	Non-ribosomal peptide biosynthesis thioesterase BasH	100/100	100/100	100/100	100/100	100/100	Not found	96/100
	*basI*	Acinetobactin biosynthesis protein BasI	97/98.8	97/98.8	97/98.8	97/98.8	97/98.8	Not found	93/98.8
	*basJ*	Acinetobactin biosynthesis protein BasJ	98/100	98/100	98/100	98/100	98/100	Not found	98/100
	*entE*	Non-ribosomal peptide synthetase EntE	100/100	100/100	100/100	100/100	100/100	100/100	96/100
	*bauA*	TonB-dependent siderophore receptor BauA	99/100	99/100	99/100	99/100	99/100	99/100	99/100
	*bauB*	Ferric siderophore ABC transporter, periplasmic siderophore-binding protein BauB	100/100	100/100	100/100	100/100	100/100	100/100	97/100
	*bauC*	Ferric siderophore ABC transporter, permease protein BauC	98/100	98/100	98/100	98/100	98/100	98/100	95/83.1
	*bauD*	Ferric siderophore ABC transporter, permease protein BauD	99/100	99/100	99/100	99/100	99/100	99/100	96/99.9
	*bauE*	Ferric siderophore ABC transporter, ATP-binding protein BauE	100/100	100/100	100/100	100/100	100/100	100/100	99/100
	*bauF*	Siderophore-interacting protein	100/100	100/100	100/100	100/100	100/100	100/100	97/100

^1^ Values represent identities and coverages of nucleotide sequence comparison between our isolates and the reference *A. baumannii* ACICU. The gray color indicates 100% identity to the reference in both identity and coverage. ^2^ PNAG: Poly-beta-1,6-N-acetyl-D-glucosamine.

## Data Availability

Data are provided in the article and Supplementary Materials.

## Supplementary Materials

The following are available online at https://www.mdpi.com/article/10.3390/microorganisms9061295/s1. Table S1. Oligonucleotide sequences used in this study. Table S2. Generation time of colistin- and carbapenem-resistant *Acinetobacter baumannii* isolates.
