# Supplementary material for: Antimicrobial Resistance Mechanisms and Virulence of Colistin- and Carbapenem-Resistant Acinetobacter baumannii Isolated from a Teaching Hospital in Taiwan
Source: Microorganisms. 2021 Jun 14;9(6):1295. doi: 10.3390/microorganisms9061295 (PMC8232278; doi:10.3390/microorganisms9061295)
Supplement: Supplementary file 1 [file microorganisms-09-01295-s001.zip › microorganisms-1252181-supplementary.pdf]

Table S1. Oligonucleotide sequences used in this study.

Primer	Primer sequence (5' to 3')	Reference
Species identification		
D14	GACAACAGTTATAAGGTTTCAGGTG	
D19	CCGCTATCTGTATCCGCAGTA	
D16	GATAACAGCTATAAAGTTTCAGGTGGT	
D8	CAAAAACGTACAGTTGTACCACTGC	[1]
Sp2F	GTTCCTGATCCGAAATTCTCG	
Sp4F	CACGCCGTAAGAGTGCATTA	
Sp4R	AACGGAGCTTGTCAGGGTTA	
Oxacillinase genes		
bla_{OXA-51}-F	TAATGCTTTGATCGGCCTTG	
bla_{OXA-51}-R	TGGATTGCACTTCATCTTGG	
bla_{OXA-58}-F	AAGTATTGGGGCTTGTGCTG	
bla_{OXA-58}-R	CCCCTCTGCGCTCTACATAC	[2]
bla_{OXA-23}-F	GATCGGATTGGAGAACCAGA	
bla_{OXA-23}-R	ATTTCTGACCGCATTTCCAT	
bla_{OXA-24}-F	GGTTAGTTGGCCCCCTTAAA	
bla_{OXA-24}-R	AGTTGAGCGAAAAGGGGATT	
pmrCAB amplification for Sanger sequencing		
pmrC-F	CGGTAAGCGTGATACCTTTGA	
pmrC-IF	CCGTGGTCGGTGTTTTACTT	
pmrC-R	GAGCCAAACCATCTAAACCGT	
pmrA-F	ACTGGACATGTTGCACTCTT	
pmrA-R	TGAAGTGCAACCTTATAAGCAC	[3]
pmrB-F	ATTCGAACCATCCGAGGACT	
pmrB-R	TGCGAGGAGCACATTTTCTA	
lpxA-F	TGAAGCATTAGCTCAAGTTT	
lpxA-R	GTCAGCAAATCAATACAAGA	
lpxD-F	CAAAGTATGAATACAACTTTTGAG	
lpxD-R	TTGAGCTAATGCTTCAACAA	[4]
lpxC-F	TGAAGATGATGTTCCTGCAA	
lpxC-R	TGGTGAAAATCAGGCAATGAA	
eptA gene amplification		
eptA1-F	CTCAAGGAGCCATCGTCATAT	
eptA1-R	GGGTTACGACTGCCCTCTTA	[5]
eptA2-F	AGTTTTACTAGCTAAGATTTCGAG	

Primer	Primer sequence (5' to 3')	Reference
eptA gene amplification		
eptA2-R	TGATGCATAGCTGACGAAG	[5]
Prom eptA-F	CAGGAAGTTCCTTTATCTGG	This study
Prom eptA-R	CTAGCCAGATAGATAAA	
Primers for qRT-PCR		
rpoB-QF	ATGCCGCCTGAAAAAGTAAC	[3]
rpoB-QR	TCCGCACGTAAAGTAGGAAC	
pmrC-QF	TGGAAATGGACTGCCAAAAT	
pmrC-QR	ACTTCCGAAACATCGGTCTG	
eptA-QF	TGCTTTTATCTATCTGGCTAGGTT	[5]
eptA-QR	TGTCGCCCCTAAGAAAAGAACT	
naxD-QF	ACCGATCAATACCCCTCATT	[6]
naxD-QR	GTTCGCCATAATTGGTCAGT	

Table S2. Generation time of colistin- and carbapenem-resistant *Acinetobacter baumannii* isolates.

Isolate	Generation time (min)
T1060317	27.65 ± 1.31
T1060361	24.65 ± 1.38
T1060578	25.49 ± 0.93
T1060580	26.62 ± 0.83
T1070171	25.76 ± 1.13
T1070213	25.03 ± 0.91
T1070678	26.48 ± 2.10
ATCC 19606	26.83 ± 1.42

References

1. Higgins, P.G.; Lehmann, M.; Wisplinghoff, H.; Seifert, H. gyrB multiplex PCR to differentiate between *Acinetobacter calcoaceticus* and *Acinetobacter* genomic species 3. *J Clin Microbiol* **2010**, *48*, 4592-4594, doi:10.1128/JCM.01765-10.
2. Woodford, N.; Ellington, M.J.; Coelho, J.M.; Turton, J.F.; Ward, M.E.; Brown, S.; Amyes, S.G.; Livermore, D.M. Multiplex PCR for genes encoding prevalent OXA carbapenemases in *Acinetobacter* spp. *Int J Antimicrob Agents* **2006**, *27*, 351-353, doi:10.1016/j.ijantimicag.2006.01.004.
3. Lee, J.Y.; Chung, E.S.; Ko, K.S. Transition of colistin dependence into colistin resistance in *Acinetobacter baumannii*. *Sci Rep* **2017**, *7*, 14216, doi:10.1038/s41598-017-14609-0.
4. Moffatt, J.H.; Harper, M.; Harrison, P.; Hale, J.D.; Vinogradov, E.; Seemann, T.; Henry, R.; Crane, B.; St Michael, F.; Cox, A.D., et al. Colistin resistance in *Acinetobacter baumannii* is mediated by complete loss of lipopolysaccharide production. *Antimicrob Agents Chemother* **2010**, *54*, 4971-4977, doi:10.1128/aac.00834-10.
5. Trebosc, V.; Gartenmann, S.; Totzl, M.; Lucchini, V.; Schellhorn, B.; Pieren, M.; Lociuro, S.; Gitzinger, M.; Tigges, M.; Bumann, D., et al. Dissecting colistin resistance mechanisms in extensively drug-resistant *Acinetobacter baumannii* clinical isolates. *mBio* **2019**, *10*, doi:10.1128/mBio.01083-19.
6. Chin, C.Y.; Gregg, K.A.; Napier, B.A.; Ernst, R.K.; Weiss, D.S. A PmrB-regulated deacetylase required for lipid A modification and polymyxin resistance in *Acinetobacter baumannii*. *Antimicrob Agents Chemother* **2015**, *59*, 7911-7914, doi:10.1128/aac.00515-15.
